# A multi-robot collaborative manipulation framework for dynamic and obstacle-dense environments: integration of deep learning for real-time task execution

**DOI:** 10.3389/frobt.2025.1585544

**Published:** 2025-07-30

**Authors:** Afnan Ahmed Adil, Saber Sakhrieh, Jinane Mounsef, Noel Maalouf

**Affiliations:** ^1^ Advanced Research and Innovation Center (ARIC), Khalifa University of Science and Technology, Abu Dhabi, United Arab Emirates; ^2^ Electrical Engineering and Computing Department, Rochester Institute of Technology, Dubai, United Arab Emirates; ^3^ Electrical and Computer Engineering Department, Lebanese American University, Byblos, Lebanon

**Keywords:** multi-robot system, cooperative object transportation, path planning, RRT∗, YOLO, dynamic obstacles

## Abstract

This paper presents a multi-robot collaborative manipulation framework, implemented in the Gazebo simulation environment, designed to enable the execution of autonomous tasks by mobile manipulators in dynamic environments and dense obstacles. The system consists of multiple mobile robot platforms, each equipped with a robotic manipulator, a simulated RGB-D camera, and a 2D LiDAR sensor on the mobile base, facilitating task coordination, object detection, and advanced collision avoidance within a simulated warehouse setting. A leader-follower architecture governs collaboration, allowing for the dynamic formation of teams to tackle tasks requiring combined effort, such as transporting heavy objects. Task allocation and control are achieved through a centralized control structure architecture in which the leader robot coordinates subordinate units based on high-level task assignments. The framework incorporates deep learning-based object detection (YOLOv2) to identify target objects using a simulated RGB-D camera mounted on the manipulator’s end-effector. Path planning is achieved through a sampling-based algorithm that is integrated with the LiDAR data to facilitate precise obstacle avoidance and localization. It also provides real-time path rerouting for safe navigation when dynamically moving obstacles, such as humans or other entities, intersect planned paths. This functionality ensures uninterrupted task execution and enhances safety in human-robot shared spaces. High-level task scheduling and control transitions are managed using MATLAB and Stateflow logic, while ROS facilitates data communication between MATLAB, Simulink, and Gazebo. This multirobot architecture is adaptable, allowing configuration of team size for collaborative tasks based on load requirements and environmental complexity. By integrating computer vision and deep learning for visual processing, and YOLOv2 for object detection, the system efficiently identifies, picks, and transports objects to designated locations, demonstrating the scalability of multi-robot framework for future applications in logistics automation, collaborative manufacturing, and dynamic human-robot interaction scenarios.

## 1 Introduction

Multi-robot systems (MRS) have gained significant attention in recent years due to their advantages over single-robot systems, including lower cost, increased robustness, and enhanced scalability. Significant efforts have been dedicated to developing MRS that display cooperative behavior, as numerous real-world complex challenges cannot be effectively addressed by a single robot due to physical constraints, limited resources, or restricted access to information. These factors underscore the critical role of collaborative behavior, with each unit contributing to the attainment of a shared objective (An et al., 2023).

A vital capability for MRS is their ability to navigate and determine both their current location and destination within a broad area of operation, a process commonly referred to as path planning. This skill requires robots to carry out core tasks, including identifying their goals and current locations, recording visited points, and addressing the primary path-planning challenge, which is finding the optimal route for the robot to reach its destination while avoiding obstacles along the way (Thabit and Mohades, 2019). The objective is for the robots to move from their starting positions within a shared environment to their destination, following the most efficient route and avoiding both other robots and any obstacles in their path. Executing task allocation and path planning for scenarios like order picking is complex and involves multiple constraints (Kyprianou et al., 2021). Accomplishing these tasks, especially within dynamically changing real-world environments, requires meticulous design and high levels of detail. This necessity arises because robots must not only determine the optimal path to their targets but also navigate a landscape filled with various obstacles, such as people and walls, while accounting for any physical access limitations present in the environment.

Path planning algorithms are categorized into three main types: search-based, heuristic-based, and sampling-based (Wong et al., 2023). The A ∗ algorithm is a prominent example of search-based path planning, where it first models the environment and then identifies target nodes to minimize inefficient exploration, effectively seeking optimal solutions (Hart et al., 1968). However, A ∗ is computationally demanding in high-dimensional spaces or large environments, leading to significant increases in processing time. In contrast, heuristic-based path planning algorithms, such as Ant Colony Optimization (ACO) and Artificial Potential Field (APF), iteratively explore environments using functions to refine solutions. However, their convergence rates can be inconsistent (Kyprianou et al., 2021). Due to their high computational requirements, both search-based and heuristic-based path planning methods are often unsuitable for real-time applications.

Sampling-based path planning algorithms, including the probabilistic roadmap (Kavraki et al., 1996) and Rapidly-exploring Random Tree (RRT) (LaValle, 1998), offer distinct advantages for real-time use. The probabilistic roadmap algorithm simplifies environment modeling by scattering sample points across space, effectively covering key features and enabling path planning in high-dimensional environments. The RRT algorithm builds on sampling methods by randomly expanding nodes to explore the environment more thoroughly. While the basic RRT algorithm can identify a viable path through the search space relatively quickly, it encounters challenges, such as spending excessive time in unproductive regions. To address these limitations, various improvements have been proposed, leading to the development of the RRT ∗ algorithm. RRT ∗ is one of the most widely applied sampling-based algorithms for asymptotically optimal motion planning, setting a foundation for achieving optimality across motion planning (Solovey et al., 2019). Unlike RRT, which seeks any feasible path to the goal, RRT ∗ iteratively improves the path by rewiring nodes in the tree to reduce the overall path cost, ensuring that the path converges toward an optimal solution as more nodes are added. This characteristic makes RRT ∗ well-suited for applications requiring efficient, high-quality path planning in complex, high-dimensional environments (Wong et al., 2023).

MRS architectures generally fall into two categories: centralized and decentralized (An et al., 2023). In a centralized setup, a lead robot collects data from others, makes decisions, and sends control commands to the rest, which follow the assigned tasks. While this allows for centralized control, it is prone to single-point failures that can compromise system reliability and limit scalability due to bandwidth and computational constraints. Decentralized architectures, however, provide each robot with full autonomy, enabling them to communicate directly with one another without a central monitoring node. This setup enhances system robustness, as individual robot failures do not impact the whole system, though maintaining synchronization and coherence in dynamic environments presents a challenge (Jawhar et al., 2022).

In collaborative object transportation tasks for MRS, it is crucial to detect obstacles and identify transportable objects in real time rapidly. State-of-the-art computer vision models typically rely on deep learning, which often results in computationally heavy methods unsuited for real-time operation on portable devices (Queralta et al., 2020). To address this, recent research has emphasized developing lightweight, faster models capable of real-time performance with limited hardware resources. One approach, semantic segmentation, assigns each image pixel a class label, grouping adjacent labeled pixels to form recognizable objects (Lateef and Ruichek, 2019). Although this method performs well when conditions closely resemble training data, its accuracy declines in unfamiliar settings, underscoring the need for diverse training datasets and domain adaptation techniques. Another technique is object detection, which involves identifying instances of specific classes within digital images or video. However, object detection demands substantial computing power and memory to achieve real-time performance, making it challenging to balance speed with accuracy (Jiao et al., 2019). In MRS operations, algorithms must be as responsive as possible without compromising reliability. The You Only Look Once (YOLO) framework (Jiang et al., 2022) is a cutting-edge technique in real-time object detection, offering significant speed and accuracy improvements over conventional methods like Convolutional Neural Networks (CNNs). Moreover, multi-modal information fusion techniques aim to combine data from multiple sources, such as images and LiDAR, to enhance detection and positioning accuracy in collaborative MRS tasks (An et al., 2023). These algorithms integrate LiDAR scan data from each robot, creating a composite view of the environment that provides real-time and precise object localization. By merging data from different sensors, multi-modal fusion can improve the reliability of obstacle and object detection, even in complex or dynamic environments, thus enabling MRS to perform more effectively in tasks that require high responsiveness and situational awareness. Integrating such data sources also helps mitigate the limitations of each individual sensor, offering a more comprehensive perception framework essential for real-time MRS operations.

The main contributions of this work are as follows:

• A centralized multi-robot collaborative manipulation framework is proposed to enable autonomous object transportation in dynamic, obstacle-dense environments. The system employs a leader-follower control structure, where a designated leader robot dynamically allocates tasks and coordinates multiple subordinate mobile manipulators. This allows for efficient division of labor, real-time coordination, and the ability to perform collaborative tasks that exceed the capability of a single robot.
• The framework integrates deep learning-based object detection (YOLOv2) with sampling-based path planning (RRT ∗ ) and LiDAR sensing to achieve accurate perception and responsive navigation. The YOLOv2 model, deployed on an RGB-D camera mounted at the manipulator’s end-effector, enables reliable real-time object identification, while LiDAR-enhanced RRT ∗ planning ensures dynamic obstacle avoidance through real-time path rerouting. This tight coupling of perception and motion planning supports safe, autonomous operation in unpredictable human-robot environments.
• The system demonstrates high scalability and adaptability through extensive simulation in both 2D and 3D environments, supporting synchronized operation among multiple HUSKY-KINOVA robot pairs. The framework successfully handles complex logistics tasks, including the cooperative transport of large objects, while maintaining formation and avoiding collisions. These capabilities underline the framework’s relevance for real-world applications such as warehouse automation, smart manufacturing, and collaborative human-robot systems.

The structure of this paper is organized as follows: Section 2 presents a comprehensive review of related work, discussing recent advancements in multi-robot collaborative object transportation tasks, real-time object detection, and path planning algorithms. Section 3 details the proposed methodology, outlining the multi-robot framework architecture, control strategies, object detection integration, and path planning technique used for effective task execution in dynamic environments. Section 4 provides an analysis of the results, demonstrating the framework’s performance and discussing its effectiveness in handling a complex collaborative task. Finally, Section 6 summarizes key findings, highlights the implications of the framework for future applications, and suggests areas for further research.

## 2 Literature review

One prominent application of MRS is cooperative object transport (COT), which holds great promise in practical scenarios. COT focuses on how teams of robots collaborate to move heavy objects that a single robot would be unable to transport on its own (Farinelli et al., 2017). An earlier related research field is the multi-robot box-pushing case, where several robots must collaborate to move a box from one location to another (Ghosh et al., 2012). Following this, cooperative object transport was applied in structured environments with static obstacles, such as warehouse environments (Bechlioulis and Kyriakopoulos, 2018). More recent studies have shifted the focus to COT systems operating in unstructured and dynamic environments (Farivarnejad et al., 2021).

In Bechlioulis and Kyriakopoulos (2018), the authors proposed a system that tackles the challenge of COT in a workspace constrained by static obstacles, where coordination among robots is achieved through implicit communication via the commonly grasped object. The system follows a leader-follower architecture, where the leader has knowledge of the locations of the obstacles. On the other hand, the follower estimates the object trajectory using a proposed estimation algorithm by observing the object’s motion without directly communicating with the leader. However, the approach has certain limitations. It does not account for environments with dynamically moving obstacles, and has uncertainties in the grasped object’s model. Additionally, the framework has only one leader robot, which reduces the system’s robustness against potential faults. Building on this work, the authors proposed an approach that coordinates the transportation of a rigidly grasped object by multiple mobile manipulators within a compact planar workspace that includes arbitrarily shaped obstacles in (Vlantis et al., 2022). Unlike the previous work, this method accounts for the dynamic configuration changes caused by object rotation and manipulator movement. The high-level planner constructs a sequence of adjacent configuration space cells that connect the system’s initial and target configurations, ensuring a safe path for the entire system. However, the scheme is limited to 2D environments and only considers static obstacles.

Choi and Kim (2021) introduced a decentralized approach for MRS operations aimed at achieving efficient collaborative object transportation in challenging terrains. Their proposed method leverages fuzzy inference systems, optimized through a Genetic Algorithm (GA), to provide near-optimal navigation solutions in unstructured environments. Experimental results demonstrated that the MRS, utilizing these well-trained fuzzy inference models, was able to successfully transport objects to target locations while minimizing travel distance and avoiding collisions. However, the proposed system does not account for several practical factors, such as system dynamics and mission execution time.

Koung et al. (2021) proposed a method for COT with obstacle avoidance, based on an optimization approach. The object avoidance tasks were formulated as equality or inequality constraints, which were solved using a Hierarchical Quadratic Programming (HQP) method (Kanoun et al., 2011). In this approach, an object is placed on top of a group of non-holonomic mobile robots without the need for grasping or caging techniques. The authors defined the formation task using inter-distances between robots to maintain a rigid shape, with the formation easily adaptable to the object’s shape by adjusting the inter-distance definitions. The proposed approach was validated through simulations in Gazebo and ROS, as well as actual experiments with HUSKY robots. However, the transition process between different states in the system is managed by a state machine, which updates the state based on a designer-specified metric. As a result, the system’s reliability is highly dependent on the operator’s knowledge and the sensitivity of the sensors used.

Juang et al. (2022) proposed a novel multistage evolutionary fuzzy control configuration for navigating multi-robots that cooperatively transport an overhead object in unknown scenarios. Their approach utilizes a leader-follower mechanism to coordinate the movement of robots carrying an object placed overhead, without physical attachment to the robots, distinguishing it from traditional transportation methods. Unlike map-based navigation schemes, this article addresses the challenge of map-free navigation for multiple robots in environments where terrain and obstacles are unfamiliar. However, a significant limitation arises from the need to account for the dynamic movement of the carried object, which can restrict robot mobility and complicate multi-robot control tasks.

Yi et al. (2025) presented a dynamic collaboration framework for heterogeneous robots in multi-agent pickup and delivery tasks. The approach pairs mobile manipulators and transport agents to execute tasks more efficiently through an auction-based algorithm and partial trajectory planning. Instead of computing complete paths in advance, the system plans segments and reassigns pairs through an auction algorithm. This flexible framework enables robots to form temporary teams and execute tasks cooperatively based on their capabilities and real-time conditions. Simulations show it performs better than a baseline without dynamic pairing. However, the framework assumes uniform capabilities within each robot group. Additionally, this method is designed for robots that handle lightweight objects individually and is not suitable for heavy items that require multiple robots to carry together.

Muhammed et al. (2024) introduced a decentralized Model Predictive Control (MPC) approach for cooperative object transportation with MRS. The primary objective is to enable robots to make decisions individually and in real-time, while still optimizing their actions collectively toward a shared task. The framework effectively handles communication limits and changing conditions on the fly, allowing robots to respond promptly. Simulations demonstrate that this method performs well under normal conditions and maintains stability when faced with uncertainties. However, the approach may become less effective in large formations or in highly restrictive spaces due to growing computational complexity, which can affect real-time performance. Furthermore, scaling the method to a large number of robots may undermine its robustness.

Kennel-Maushart and Coros (2024) presented a method designed for differential-drive robots to collaboratively transport an object through a constrained environment. The control framework is decentralized, allowing each robot to make decisions based on local information while exchanging messages with its teammates, thereby improving robustness and scalability. The approach is formulated as a bi-level optimization problem. At the lower level, it calculates the distribution of normal forces on the wheels to maintain stability and avoid tip-over. At the upper level, it plans trajectories that satisfy non-holonomic constraints and guide the robots toward their destination in a coordinated way. The method utilizes direct transcription optimization, solving trajectory and control signals together in a unified framework. Using this approach, the robots can navigate around static obstacles and execute complex maneuvers collectively, as demonstrated in experiments with Clearpath Husky robots. Nonetheless, the framework presupposes knowledge of the object’s properties and the environment’s configuration. Additionally, it ignores dynamic effects, such as inertia and momentum, which may affect performance in challenging scenarios.

Path planning is essential for COT, with the primary objective of any path planning algorithm being to establish a collision-free route from a starting position to a target position within the robot configuration space. Probabilistic planning algorithms, like the RRT, offer quick solutions. Since its introduction, RRT has become one of the most widely used probabilistic planning algorithms due to its speed and simplicity, incrementally expanding a tree in the configuration space until reaching the goal (Connell and La, 2018). An enhanced version, the RRT ∗ algorithm, significantly improves RRT’s solution optimality and has demonstrated an asymptotically suboptimal solution, balancing speed with improved path quality (Chao et al., 2018). In V Y et al. (2024), the authors presented an innovative path planning approach for unmanned ground vehicles in disaster relief scenarios by integrating the advanced capabilities of RRT ∗ algorithms with YOLOv4, a cutting-edge object detection method for optimizing human detection. As an exploratory algorithm, their proposed system is highly effective at navigating unfamiliar environments without requiring a complete map, enabling it to plan safe paths around obstacles and hazards. This adaptability makes it particularly well-suited for dynamic, unpredictable environments typical of disaster relief operations.

Building on the insights from the above discussion and specifically from the results demonstrated in (V Y et al., 2024) for single-robot systems, we propose a multi-robot mobile manipulator system designed to operate in dynamic environments. This system integrates advanced robotic control and detection capabilities for effective object identification and categorization. Equipped with an RGB-D camera on the manipulator’s end-effector, it utilizes YOLO-based object detection, while a 2-D lidar sensor mounted on the robot’s base enables localization through a pre-computed map. The mobile robot’s path planning subsystem is triggered and plans a path using the RRT ∗ algorithm based on a binary occupancy grid map. This configuration, with trained YOLO models, ensures that object detection and path planning can adapt to changes within dynamic environments, enhancing the system’s reliability and versatility. To further clarify the distinctions between the proposed framework and existing methods, Table 1 provides a comparative summary of related multi-robot collaborative manipulation approaches. This comparison underscores the unique contributions of the presented work, which integrates a leader-follower control architecture, deep-learning–based object detection, and the RRT* path planning algorithm to enable dynamic obstacle avoidance while offering a scalable, adaptable, and real-time collaborative solution.

**TABLE 1:** Comparison of related work in multi-robot collaborative manipulation frameworks.

Reference	Control	Obstacles	Object	Robotic	MRS	Path	Simulation/
	Architecture		Detection	Manipulator		Planning	Experiment
Bechlioulis and Kyriakopoulos (2018)	Decentralized	Static	✗	✓	✓	APF	Simulation (2D and 3D)
Vlantis et al. (2022)	Decentralized	Static	✗	✓	✓	Config-space	Simulation (2D)
Choi and Kim (2021)	Decentralized	Static	✗	✗	✓	GA	Simulation (2D)
Koung et al. (2021)	Centralized	Static	✗	✗	✓	HQP	Both
Juang et al. (2022)	Centralized	Static	✗	✗	✓	Fuzzy Control	Both
Yi et al. (2025)	Decentralized	Static	✗	✓	✓	${\text{A}}^{*}$	Simulation (3D)
Muhammed et al. (2024)	Decentralized	Dynamic	✗	✗	✓	MPC	Simulation (2D)
Kennel-Maushart and Coros (2024)	Decentralized	Static	✗	✓	✓	Direct Transcription	Both
V Y et al. (2024)	Centralized	Dynamic	YOLO	✗	✗	${\text{RRT}}^{*}$	Simulation
This work	Centralized	Dynamic	YOLO	✓	✓	${\text{RRT}}^{*}$	Simulation (2D and 3D)

## 3 Methodology

The proposed multi-robot collaborative manipulation system operates in a simulated environment, enabling autonomous task execution in dynamic spaces with variable obstacles and human interference. This section details the techniques and tools utilized for task and joint-space trajectory generation, object detection, path planning, and collaborative control, all implemented within a Gazebo-based simulation environment.

**FIGURE 1:**
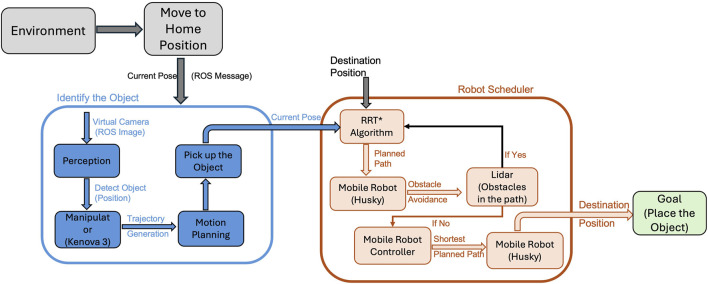
Workflow of the proposed multi-robot collaborative manipulation framework.

Figure 1 illustrates the high level end-to-end workflow of the multi-robot collaborative manipulation framework. The process begins with Environment Setup, where robots move to their predefined home positions within the workspace. This ensures proper initialization and alignment of sensors before any task execution. Once in position, the Object Detection module activates, leveraging a DNN to process images from an RGB-D camera mounted on the KINOVA Gen3 manipulator’s end-effector to identify and locate objects in the environment. Detected object coordinates are then fed into the Path Planning module, which employs the RRT* algorithm integrated with LiDAR data for real-time obstacle avoidance and path rerouting. Finally, the Control Execution phase utilizes Pure Pursuit control to guide the HUSKY mobile robot along the planned trajectory while maintaining synchronization with all collaborating robots. This modular structure ensures seamless integration of perception, planning, and control, enabling efficient task execution in dynamic and obstacle-dense environments.

### 3.1 Robotic platform and kinematics

In this setup, the CAD model of the KINOVA Gen3 manipulator is imported into the MATLAB workspace as a rigid body tree (RBT) structure, forming the basis for simulating and controlling the robotic arm’s kinematic behavior. This RBT model enables accurate motion simulation by defining each joint, link, and frame of the manipulator, preserving its real-world configuration and ensuring compatibility with MATLAB’s Robotics System Toolbox.

**FIGURE 2:**
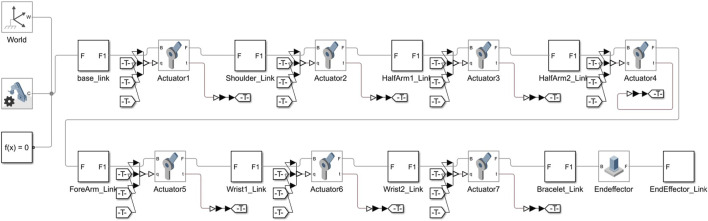
Model of Kinova robotic manipulator represented as an RBT structure, showing the sequential linkage from the base to the end-effector.

The kinematic configuration and parameters of the manipulator are implemented in Simulink using Simscape Multibody blocks. The Mechanism Configuration block specifies critical parameters such as gravity and simulation configurations for the RBT model. Within the mechanism, the configuration block defines parameters for numerical differentiation, including perturbation values for computing partial derivatives and iteration parameters for handling joint mode transitions. In Simscape, this configuration ensures that all components are subject to consistent gravity conditions, with only one such configuration block permitted per Simscape Multibody network. In the absence of this block, the model assumes a zero-gravity environment, which could compromise accuracy in realistic simulations. Figure 2 shows the used Kinova robotic manipulator model.

Each joint of the manipulator is represented as a Revolute Joint block from Simscape, which allows a single degree of freedom, restricting movement to rotation about the z-axis of the joint’s base frame. This configuration enables precise control over each joint’s rotation in line with the robot’s kinematic constraints. The relationship between the joint angles $\Theta_{i}$ and the end-effector position $P_{ee}$ can be expressed using the forward kinematics in Equation 1:

$$P_{ee}=f\left(\theta_{1},\theta_{2},\ldots ,\theta_{n}\right)$$ (1)

where $P_{ee}={\left[\begin{array}{ccc} x & y & z \end{array}\right]}^{T}$ denotes the position of the end-effector in the Cartesian coordinate system and f is a function mapping the joint angles to the end-effector pose.

**FIGURE 3:**
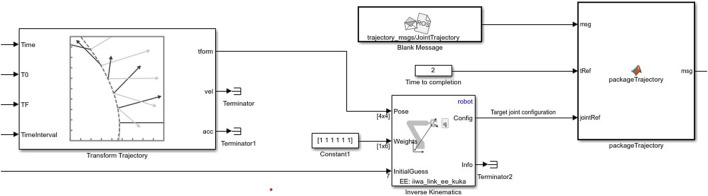
Simulink subsystem for trajectory transformation and inverse kinematics of a robotic arm.

To monitor and control the manipulator’s pose, the output of the RBT is fed to the Get Transform block, which calculates the homogeneous transformation matrix $\left(T_{\mathrm{Form}}\right)$ between selected body frames. This transformation matrix is defined in Equation 2:

$$T_{\mathrm{Form}}=\left[\begin{array}{cc} R & d\\ 0 & 1 \end{array}\right]$$ (2)

this transformation matrix, which specifies the spatial relationship between body frames, is essential for controlling the manipulator’s position relative to objects within the workspace. The Get Transform block computes transformations based on an input configuration, Config, allowing for real-time coordinate conversion from the source frame to the target frame, where R is the rotation matrix describing the orientation and d is the translation vector. The Get Transform block computes transformations based on an input configuration, Config, allowing for real-time coordinate conversion from the source frame to the target frame, as shown in Figure 3.

The Coordinate Transformation Conversion block from the Robotics System Toolbox processes $T_{\mathrm{Form}}$ and converts it into a range of coordinate representations as needed, including Axis-Angle, Euler Angles, Quaternion, Rotation Matrix, and Translation Vector. The selection of a specific coordinate system provides flexibility in configuring control inputs, thereby aligning the kinematic outputs with downstream processes in the model. Equations 3–7 define each of the considered coordinate representations:

1. Axis-Angle:

$$Axis\text{\_}Angle=\left[n,\theta \right],$$ (3)

where n is the rotation axis and θ is the rotation angle.

2. Euler Angles:

$$\left[\begin{array}{c} \phi \\ \theta \\ \psi \end{array}\right]=\left[\begin{array}{c} tan^{-1}\left(\frac{r_{21}}{r_{11}}\right)\\ sin^{-1}\left(-r_{31}\right)\\ tan^{-1}\left(\frac{r_{32}}{r_{33}}\right) \end{array}\right]$$ (4)

3. Homogeneous Transformation:

$$T_{\mathrm{Form}}=\left[\begin{array}{cc} R & p\\ 0 & 1 \end{array}\right]$$ (5)

4. Quaternion:

$$q=\left[\begin{array}{c} w\\ x\\ y\\ z \end{array}\right]=\left[\begin{array}{c} cos\left(\frac{\theta }{2}\right)\\ n_{x}sin\left(\frac{\theta }{2}\right)\\ n_{y}sin\left(\frac{\theta }{2}\right)\\ n_{z}sin\left(\frac{\theta }{2}\right) \end{array}\right]$$ (6)

5. Translation Vector:

$$TrVec=\left[\begin{array}{c} x\\ y\\ z \end{array}\right]$$ (7)

The Inverse Kinematics (IK) subsystem orchestrates the manipulator’s motion by calculating the required joint configurations for reaching a given end-effector pose. This subsystem uses the IK block, which employs an IK solver with the RBT model to achieve a specified target pose. This IK block takes as inputs the end-effector’s desired pose, a tolerance weight, and an initial joint configuration guess, outputting a Config that meets the end-effector pose constraints within specified tolerances. This subsystem is crucial for enabling the dynamic motion of the manipulator towards detected objects, as shown in Equation 8:

$$Config=g\left(P_{\mathrm{desired}}\right),$$ (8)

where g is the IK mapping function, taking the desired end-effector position $P_{\mathrm{desired}}$ and returning the corresponding joint angles.

The trajectory of the manipulator is informed by the position of detected objects, derived from camera inputs via a trained YOLOv2 object detector. The detector calculates object positions, which feed into the IK subsystem, enabling real-time trajectory adaptation based on object locations within the workspace.

### 3.2 Mobile robotic platform and ROS integration

**FIGURE 4:**
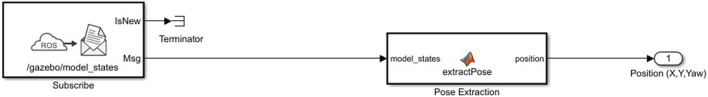
Real-time position updates and control outputs using ROS communication via Simulink Subscribe and Publish blocks.

The CAD model of the HUSKY mobile robot is imported into MATLAB as an RBT model, permitting its kinematic analysis and control. For real-time position updates, the ROS interface is employed using the Subscribe block, which receives position data as ROS messages on specific topics. This data transforms Simulink bus signals, allowing direct integration into the control logic of the robotic system, as illustrated in Figure 4.

Position updates and control outputs are disseminated to the ROS network using the Publish block. This block translates Simulink bus signals into ROS messages and transmits them on designated topics, enabling seamless two-way communication between the simulation and any real or simulated ROS environment. This integration facilitates real-time feedback for controlling the HUSKY robot, crucial for adaptive navigation and alignment tasks in unstructured or dynamic environments.

### 3.3 Object detection system

**FIGURE 5:**
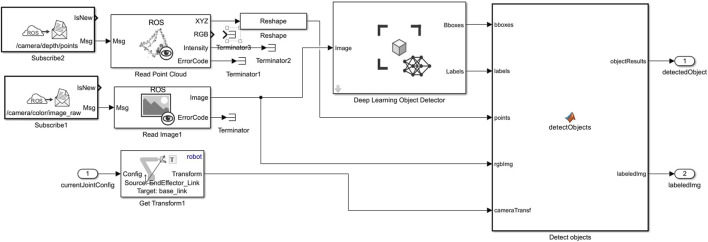
Simulink model representing object detection pipeline using a combination of ROS messages and deep learning.

Object detection is integral to the robotic system’s adaptive capabilities, specifically for identifying and locating objects in the workspace. A virtual camera sensor mounted on the end-effector, connected via ROS, streams images to MATLAB, where they are processed in real-time. The Subscribe block is used to capture these camera feeds as ROS image messages. The incoming messages are processed by the Read Image block from the ROS Toolbox, which extracts images from ROS Image or CompressedImage messages. The Read Image block converts the ROS message into a Simulink-compatible image format, facilitating seamless integration with the downstream object detection pipeline. As depicted in Figure 5, this seamless integration enables real-time image processing for object detection through the ROS interface.

The extracted images are passed to the Deep Learning Object Detector block from the Computer Vision Toolbox. This block, configured with a pre-trained YOLOv2 object detector, identifies objects in the frame, generating bounding boxes, class labels, and confidence scores for each detected object. The trained YOLOv2 model was chosen for its proven accuracy and efficiency in real-time object detection tasks. Configurable through Simulink, this block provides a straightforward interface for loading and managing the object detection model, and the block’s graphical parameters align closely with the detect function parameters in MATLAB. By enabling key parameters of the YOLOv2 object detector, such as detection thresholds, this setup achieves high-accuracy object identification and localization.

While the latest version of the YOLO architecture is YOLOv11, this study utilizes YOLOv2 due to its suitability for the specific requirements of our multi-robot collaborative manipulation framework. The primary rationale for selecting YOLOv2 lies in the nature of the simulation environment, task requirements, and computational constraints.

• Simplified Task Requirements: In the Gazebo simulation environment, the number of objects requiring detection is relatively limited compared to more complex real-world scenarios. This reduces the necessity for the advanced features and increased complexity of newer object detection frameworks such as YOLOv11. YOLOv2 provides sufficient accuracy for identifying the small set of target objects within the simulated warehouse environment, making it an appropriate choice for our application.
• Computational Efficiency and Real-Time Processing: YOLOv11 introduces significant architectural advancements over YOLOv2, including enhanced feature extraction capabilities and improved performance in detecting a wide variety of objects. However, these improvements come at the cost of higher computational complexity, larger model size, and increased resource demands. Given the constraints of our simulation setup—running Simulink models, managing path planning, collision avoidance, and motion control for up to 8 robots (4 HUSKY-KINOVA pairs) on an NVIDIA RTX 3070 GPU—YOLOv2’s lighter architecture ensures lower latency and smoother operation. Its balance of speed and accuracy is ideal for maintaining real-time performance under these computational constraints.
• Scalability and Future Enhancements: Although YOLOv2 is employed in the current framework, the modular design of our system allows for seamless upgrades to more recent object detection architectures like RF-DETR or YOLOv11, or even custom-developed DNNs in future implementations. For physical deployment, we plan to develop a lightweight custom DNN leveraging techniques such as Flash Attention for object detection. This approach will ensure even lower latency and improved performance tailored specifically to the constraints of embedded hardware systems.

In summary, YOLOv2 was chosen for its simplicity, efficiency, and ability to meet the real-time demands of our simulation environment without compromising accuracy. Its lower computational overhead and smaller model size make it ideal for our resource-constrained setup involving multiple robots and simultaneous processes.

The object coordinates are then fed into the IK subsystem, informing the trajectory planning and control of both the KINOVA manipulator and HUSKY robot, ensuring the robotic system can dynamically respond to object positions detected within the environment.

In this research framework, we have integrated six pairs of HUSKY mobile robots and KINOVA Gen3 manipulators, each operating within their designated ROS nodes. This architecture facilitates independent operation while enabling coordinated control through a unified Simulink model. By employing the Subscribe block in Simulink, we can receive real-time position and state information from each robot’s respective ROS node. The subscription allows for continuous monitoring of the kinematic states of both the HUSKY and KINOVA systems, with position vectors $P_{i}$ for the $i_{th}$ robot defined in Equation 9:

$$P_{i}=\left[\begin{array}{c} x_{i}\\ y_{i}\\ \theta_{i} \end{array}\right],$$ (9)

where $x_{i}$ and $y_{i}$ denote the Cartesian coordinates and $\theta_{i}$ represents the orientation of the $i_{th}$ robot.

**FIGURE 6:**
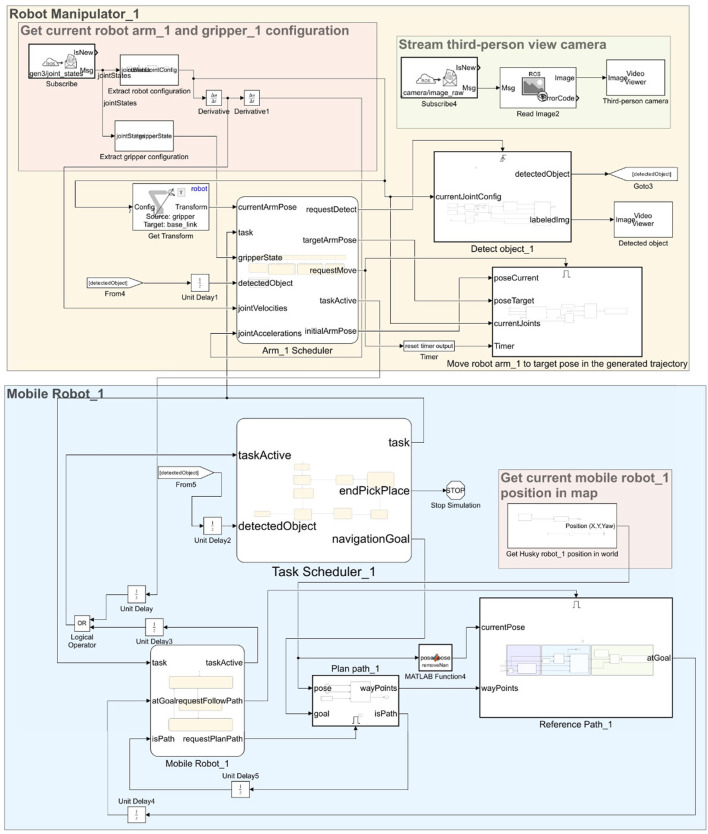
Model showcasing the Robotic Manipulator and Mobile Robot components for coordinated operations with motion planning and path following subsystems.

The command outputs are sent back to each robotic entity through the Publish block, which facilitates the transmission of control signals encoded as ROS messages. Each command can be expressed in Equation 10:

$$C_{i}=\left[\begin{array}{c} v_{i}\\ \omega_{i} \end{array}\right],$$ (10)

where $v_{i}$ is the desired linear velocity and $\omega_{i}$ is the angular velocity for the $i_{th}$ robot. This architecture allows for distinct control strategies tailored to the individual dynamics of each robotic unit, enhancing the system’s overall flexibility and responsiveness. As shown in Figure 6, the model showcases the coordinated operations of the robotic manipulator and mobile robot components, integrating motion planning and path-following subsystems.

The communication between the KINOVA manipulators and HUSKY mobile robots is facilitated via a publish-subscribe architecture established within the ROS framework. Each robot’s ROS node operates on its communication topic, enabling efficient data exchange. For instance, the HUSKY nodes publish their position data on the topic /husky_x/pose , while the KINOVA nodes publish joint state information on /kinova_x/joint_states . This segregation of topics minimizes data collision and enhances the robustness of inter-robot communication.

### 3.4 HUSKY working principle: controller, path following, and ROS integration

**FIGURE 7:**
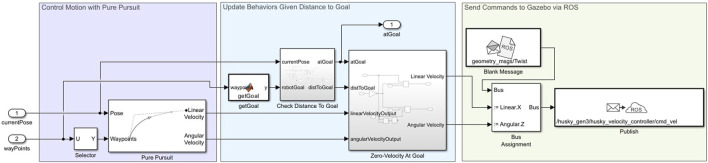
Closed-loop control for the HUSKY robot, enabling real-time path-following and obstacle avoidance in Gazebo through ROS integration.

The HUSKY robot operates under a closed-loop control system in which the controller receives real-time data regarding the robot’s pose and laser scan readings from the Gazebo environment through ROS messages. These inputs are processed via the Subscribe block in Simulink, while velocity commands are generated and sent back to the Gazebo simulation through the Publish block, which allows for seamless path-following and obstacle avoidance within the simulated environment. As shown in Figure 7, this closed-loop control ensures efficient path-following and obstacle avoidance through real-time ROS integration with Simulink.

To initialize the ROS network, rosinit (from the ROS Toolbox) is executed in MATLAB. This command establishes a local ROS master at http://localhost: 11311, ensuring smooth communication with all nodes within the ROS network.

#### 3.4.1 Input processing for localization and mapping

The Inputs subsystem within Simulink is responsible for gathering and processing the necessary input data for the control algorithm. Two main ROS subscribers are configured:

1. Velocity Subscriber: Subscribes to the /geometry_msgs/Twist topic, from which laser scan data is extracted. This 2D lidar sensor data, located on the front of the mobile base, is used to map out the robot’s environment. The lidar data provides the scan ranges and angles necessary for localization, enabling the robot to accurately position itself within a precomputed map. Mathematically, each laser scan can be represented as a series of points, as in Equation 11:

$$L={\left\{\left(r_{i},\theta_{i}\right)\right\}}_{i=1}^{n},$$ (11)

where $r_{i}$ is the range to an obstacle, and $\theta_{i}$ is the angle of detection for each point i .

2. Pose Subscriber: Subscribes to the /gazebo/model_statestopic , from which it retrieves the robot’s pose as (x,y) coordinates and the yaw angle θ . This pose information forms a vector: $P={\left[\begin{array}{ccc} x & y & \theta \end{array}\right]}^{T}$ . This vector is crucial for determining the robot’s orientation and position on the map, allowing for precise path following.

#### 3.4.2 Path specification and following

The desired path is specified through a set of waypoints, represented as an N×2 array where N is the number of waypoints in the form [x,y] . For path-following control, the Pure Pursuit algorithm is implemented via the Pure Pursuit block from the Robotics System Toolbox, designed to compute linear and angular velocity commands to guide the robot from one waypoint to the next.

1. Velocity and Heading Calculation: Using the Pure Pursuit algorithm, the linear and angular velocity commands are calculated to align the robot’s movement with the desired path. Given the robot’s current position $P=\left[\begin{array}{ccc} x & y & \theta \end{array}\right]$ and the upcoming waypoint, the algorithm computes an instantaneous target direction or “look-ahead” point on the path.
2. The Look-Ahead Distance parameter determines how far ahead on the path the robot should aim. It adjusts the angular velocity command by positioning a local goal, creating a smoother or more precise path trajectory, as in Equation 12:

$$\omega =\frac{2vsin\left(\alpha \right)}{L},$$ (12)

where v is the linear velocity, α is the heading angle to the look-ahead point, and L is the distance between the robot’s current location and the look-ahead point. This method ensures accurate path tracking by adjusting velocity commands in real time.

3. Stopping Conditions: The Check Distance to Goal and Zero-Velocity at Goal subsystems monitor the robot’s progress toward the final waypoint. When the robot reaches the goal (within a set tolerance distance), the control algorithm reduces the linear and angular velocities to zero, indicating task completion.

#### 3.4.3 Mobile robot scheduler: task coordination

The HUSKY robot’s operational states are managed by the Mobile Robot Scheduler, which consists of the following states:

1. Idle: The robot remains in an inactive state, with path planning and following deactivated.
2. PlanPath: Triggered by a command from the main task scheduler, the robot begins planning a path using the plannerRRT ∗ algorithm within a MATLAB function block. This planner calculates the optimal path on a binary occupancy grid map, providing the waypoints for navigation. The path W can be represented in Equation 13:

$$W={\left\{\left(x_{i},y_{i},\theta_{i}\right)\right\}}_{i=1}^{N}$$ (13)

3. FollowPath: After a valid path is generated (indicated by a true value of isPath ), the scheduler initiates the path-following subsystem, utilizing the Pure Pursuit controller to align the robot’s trajectory with the computed waypoints.

**FIGURE 8:**
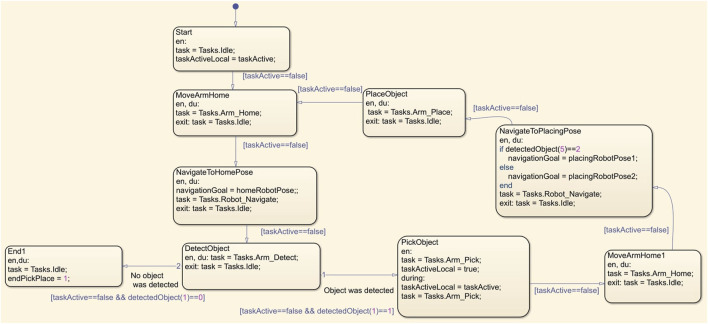
Flowchart illustrating robot’s path planning and following process from idle to execution using RRTStar and Pure Pursuit algorithms.

As illustrated in Figure 8, the Mobile Robot Scheduler coordinates the robot’s operational states, including Idle and PlanPath, to manage task execution effectively.

### 3.5 Object detection and collision avoidance in a dynamic multi-robot environment

For effective initial testing and validation, we tested the same dynamic object detection and avoidance algorithm tailored for a multi-robot system in a 2D simulation environment. This 2D simulation scenario allowed us to prototype and refine the object detection and collision avoidance algorithms before deploying them in the 3D Gazebo simulation environment.

In this 2D setup, we utilize the Multi-Robot Environment within MATLAB and Simulink, which provides a flexible framework for prototyping in a 2D multi-robot mobile robotics scenario. The Multi-Robot Environment offers essential configurations to support initial simulations:

• Map: The default map is an empty space (i.e., all free space), allowing for an unrestricted testing environment.
• Robots: The default environment includes one point robot (with zero radius), but it can support multiple robots for testing multi-agent coordination.
• Trajectory and Sensor Display: Trajectory display is initially disabled, while sensor line plots and robot IDs are displayed by default. However, for simulations involving many robots and sensors, it is recommended to disable these visual elements to improve performance.
• Sensor and Waypoint Configurations: The environment allows for flexible customization. Waypoints, lidar, object detectors, and robot detectors can all be enabled or disabled depending on the test scenario.

**FIGURE 9:**
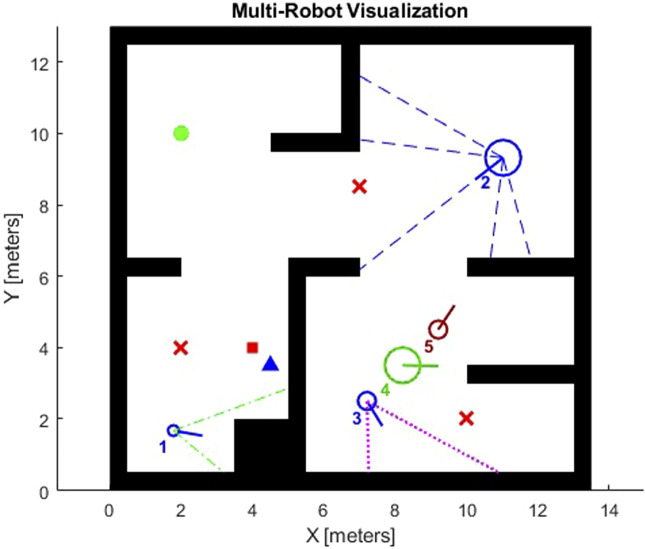
2D simulation of multi-robot object detection and collision avoidance for dynamic environments using Simulink.

Figure 9 shows the 2D simulation setup in the Multi-Robot Environment allows for effective testing and validation of dynamic object detection and collision avoidance algorithms before deployment in the 3D Gazebo simulation.

### 3.6 Implementation of multi-robot object detection and avoidance in gazebo

Following the initial testing in the 2D Multi-Robot Environment, we apply the same object detection and avoidance principles in the 3D Gazebo simulation environment. Here, we define the number of robot pairs, each comprising a HUSKY mobile robot and a KINOVA Gen3 manipulator, which are configured to work in tandem to accomplish complex tasks. Each robot pair is assigned a sequential ID, allowing us to activate, monitor, and control them individually or collectively within the environment.

Each pair of robots in Gazebo is configured to utilize a similar detection and avoidance system by subscribing to ROS topics to receive sensor data, such as laser scans and camera feeds. These inputs are then processed to detect objects dynamically and avoid collisions, enabling real-time adaptability to moving objects in the environment. The overall configuration and control strategy ensure efficient collaboration between robots, with each pair capable of handling unique subtasks while maintaining coordination across the entire fleet.

This combination of 2D and 3D simulation environments provides a robust testing workflow, ensuring that our object detection and collision avoidance algorithms are optimized for dynamic, real-time multi-robot coordination. This approach enables flexible scalability, whether coordinating a few robots or a larger fleet, ultimately improving system reliability and adaptability in real-world applications.

Our integrated coordinated system consists of six HUSKY robot and KINOVA manipulator pairs operating within the ROS framework. Each pair is identical to the configuration illustrated earlier in Figure 5. These robot pairs work collaboratively, enabling real-time coordination and adaptive collision avoidance, which ensures efficient multi-robot collaboration in dynamic and obstacle-dense environments.

## 4 Results

The experiments were conducted on a system equipped with an NVIDIA RTX 3070 GPU. The simulation setup was distributed across two environments: Gazebo, running in a virtual environment, and Simulink, operating on Windows 11. This distribution of computational resources resulted in an execution time of 1.91 s per command. However, in scenarios where obstacles were in close proximity to the robot, communication delays occasionally led to collisions. This issue highlights the impact of processing latency on real-time obstacle avoidance. Moreover, as the number of operating robots increased, the execution time grew exponentially, further exacerbating the delay. To mitigate these issues, future implementations could benefit from employing a more powerful GPU or distributing the computational load across two separate systems, one dedicated to Simulink and the other to the Gazebo environment. This will also help in deploying the algorithm on physical hardware as a stand-alone ROS node.

Our experimental setup, designed to assess the proposed multi-robot coordination, object detection, and avoidance algorithm, involved a combination of simulations in both 2D and 3D environments, progressing from simpler prototypes to more complex scenarios. In this section, we present and discuss the results obtained from the simulations, highlighting key performance metrics and insights into system behaviour in dynamic environments.

### 4.1 Object detection and avoidance performance in 2D environment

The 2D simulations provided a controlled environment to assess the accuracy and robustness of object avoidance in a multi-robot setup. The primary objectives in this environment were to validate collision-free navigation, confirm consistent waypoint following, and observe responsiveness to moving objects.

1. Detection Accuracy and Collision Avoidance: The object detection accuracy was assessed based on the percentage of moving objects correctly identified within a threshold distance of 1 m. Results show that the detection rate remained high even with multiple moving objects present, confirming the reliability of our approach in cluttered environments. The avoidance algorithm successfully prevented all collisions in scenarios involving up to 6 robots. The system showed only minimal deviation from the planned path when maneuvering around dynamic objects, confirming the effectiveness of the avoidance strategy.
2. Path Following: The robots demonstrated a high level of precision in path following, with an average error deviation of only 0.15 m from the planned trajectory. This precision is essential for close-proximity maneuvers in MRS, especially when operating in congested areas.

**FIGURE 10:**
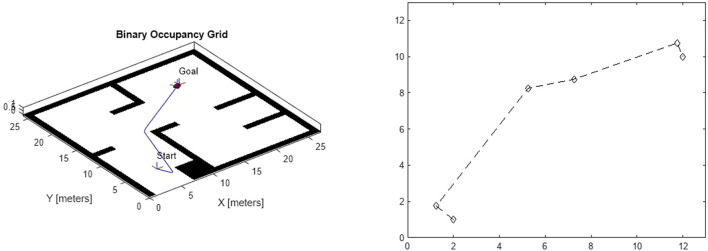
Path efficiency and precision analysis of robots in simulations with Pure Pursuit control and minimal deviation.

Figure 10 displays the 2D simulations that validated the object detection and avoidance performance, highlighting high detection accuracy, collision-free navigation, and precise path following in a multi-robot setup.

### 4.2 Multi-robot coordination and path efficiency in 3D gazebo environment

Building on the 2D results, we conducted more complex tests in the 3D Gazebo simulation environment. Here, each HUSKY and KINOVA Gen3 robot pair was tasked with navigating a dynamic environment, reacting to both static and moving objects while maintaining coordination across the fleet. Key metrics in this environment included path efficiency, computational performance, and coordination success rate.

**FIGURE 11:**
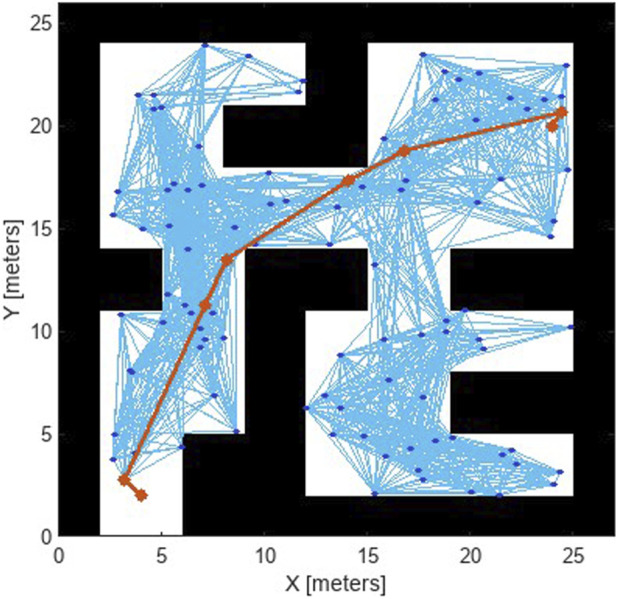
Analysis of planned path with RRT ∗ in simulations despite increased complexity.

Path efficiency was measured by the overall travel distance and time taken to reach target waypoints. Compared to the 2D environment, the 3D simulations introduced a significant increase in complexity due to terrain elevation and additional obstacles. However, the Pure Pursuit controller kept the robots within a 0.2 m threshold of the optimal path, with an average path efficiency of 92%. This efficiency demonstrates the controller’s robustness in adapting to the higher fidelity of the Gazebo environment. Figure 11 shows the analysis of the planned path using RRT ∗ .

### 4.3 Scalability and system adaptability

One of the critical objectives of our study was to evaluate the scalability and adaptability of the multi-robot coordination framework. By incrementally increasing the number of robot pairs in the Gazebo environment, we observed the following:

1. System Scalability: The results indicate that our setup can efficiently manage up to 6 HUSKY and KINOVA Gen3 pairs without degradation in performance. The modular ROS-based architecture allows for straightforward addition of new robots, demonstrating scalability in both software and hardware requirements.
2. Adaptability to Dynamic Environments: The object detection and avoidance algorithm showed resilience to variations in object speed and trajectory. Even with randomly moving objects, the robots adapted to changes and adjusted their paths effectively, demonstrating the system’s robustness in unpredictable environments.

### 4.4 Collaborative task execution: table transportation in dynamic environment

A notable experiment involved a coordinated task where four HUSKY-KINOVA pairs of robots collaborated to transport a large table from a designated start point (marked as “1”) to a destination location (marked as “2”) within the Gazebo simulation environment. The table, too large to be carried by a single robot, required each robot to grasp one corner, maintaining stable alignment while navigating through a dynamic and obstacle-filled space.

**FIGURE 12:**
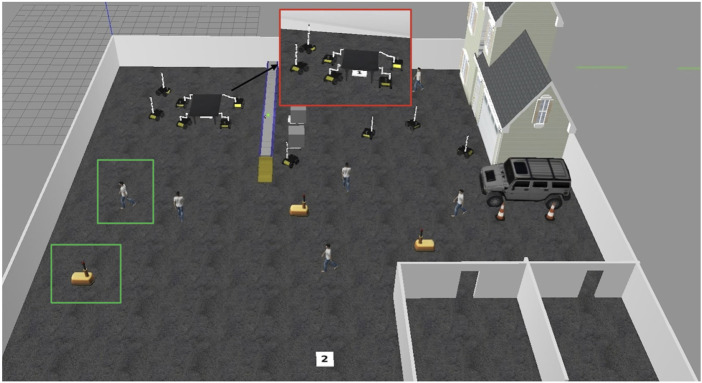
Overview of the simulated warehouse environment in Gazebo at the initial configuration. The red box highlights the coordinated HUSKY-KINOVA robot pairs transporting a large table from the start point (1) to the destination (2), while the green boxes indicate dynamic obstacles, including walking human agents and mobile robots.

**FIGURE 13:**
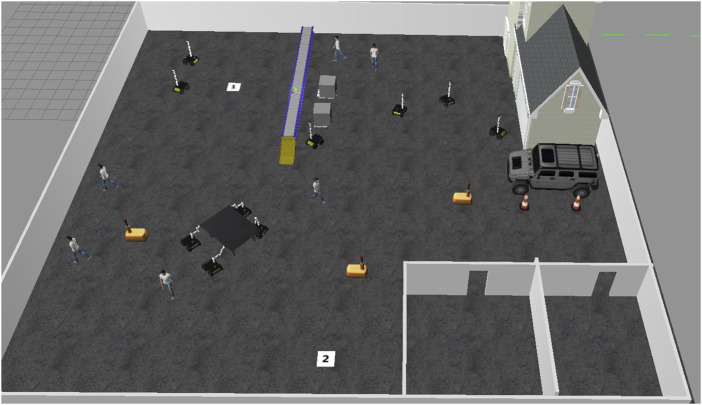
Robots performing time-synchronized obstacle avoidance, synchronized communication, and Pure Pursuit control to collaboratively transport in a dynamic environment.

**FIGURE 14:**
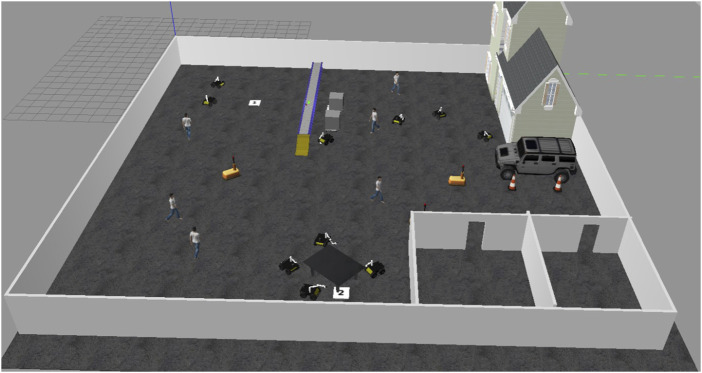
Robots performing time-synchronized obstacle avoidance, synchronized communication, and Pure Pursuit control to collaboratively transport in a dynamic environment.

1. Task Coordination and Path Planning: Each robot was positioned at a corner of the table, enabling stable transport with even weight distribution. The robots were programmed to synchronize their movements, maintaining a fixed orientation and distance from each other. The collaborative path planning module ensured that the robots operated as a cohesive unit, constantly communicating their positions and adjusting speeds through Simulink-based feedback to maintain synchronization. Figure 12 demonstrates four coordinated HUSKY-KINOVA pairs of robots ready to transport a large table from starting point “1” to destination “2” within a dynamic Gazebo simulation environment.
2. Obstacle Avoidance in a Dynamic Environment: During transportation, the robots encountered a variety of static and dynamic obstacles. As shown in Figure 12, the dynamic objects included human figures and warehouse robots moving unpredictably throughout the environment. These entities follow independent trajectories during task execution, requiring the robot pairs to perform real-time path adjustments and collision avoidance. The real-time object detection and avoidance algorithm allowed the robots to perceive and anticipate the paths of these moving entities. When an obstacle was detected along the route, the robots rerouted collaboratively, adjusting their path without breaking formation. This rerouting mechanism relied on the Pure Pursuit control block, which recalculated the path based on updated sensor data from each robot. As shown in Figure 13, the real-time object detection and avoidance algorithm enabled the robots to collaboratively reroute around dynamic obstacles while maintaining formation during the transportation task.
3. Communication and Synchronization: The robots communicated continuously through ROS messages through Simulink’s Subscribe and Publish blocks. Position data and velocity commands were exchanged in real-time to synchronize movements and avoid collisions. This communication setup ensured that each robot adjusted its motion in response to the others, aligning movements precisely to prevent the table from tilting or shifting during transportation.
4. Results of the Task Execution: The collaborative system successfully transported the table from point “1” to point “2” without collisions, showcasing robust collision avoidance and coordination even with moving obstacles. The robots maintained a stable configuration throughout, demonstrating high adaptability by rerouting effectively whenever an obstacle obstructed the planned path. This seamless adaptation highlighted the reliability of the path planning and Pure Pursuit-based control mechanisms under varying environmental conditions. Figure 14 shows the collaborative system successfully transporting the table from point “1” to point “2,” demonstrating effective collision avoidance, coordination, and adaptability in dynamic environments.

This experiment underscores the capability of the system to handle complex, cooperative tasks in a dynamic environment. The ability of the robots to reroute in real-time, coordinate effectively through communication protocols, and adjust to unpredictable obstacles demonstrates significant potential for practical applications in multi-robot logistics and transport tasks in environments like warehouses, factories, or construction sites.

## 5 Discussion

The quantitative evaluation of the proposed multi-robot collaborative manipulation framework in Table 2 highlights its effectiveness across different testing environments: 2D simulation (Test 1) and 3D Gazebo simulation (Test 2). The object detection accuracy remains high across both test scenarios, achieving 95% in 2D simulation and 92% in Gazebo. These results demonstrate the robustness of the deep learning-based detection system. While there is a slight decrease in accuracy in the more complex 3D environment, the results still indicate reliable perception performance. Collision avoidance is highly effective in 2D, achieving a 100% success rate, but slightly lower (90%) in the 3D simulation, likely due to more realistic physics and sensor noise in Gazebo. Similarly, path-following error increases from 0.15 m in 2D to 0.20 m in 3D. This reflects the added complexity of navigation in a three-dimensional space. Path efficiency also remains strong, with 94% in 2D and 90% in 3D, indicating that the system maintains near-optimal paths even with added environmental challenges. However, scalability decreases from 8 robots in 2D to 6 robots in 3D, which suggests potential computational or communication constraints in the more complex setting. Lastly, real-time adaptation was only evaluated in the 3D Gazebo simulation, where the system successfully re-routes paths in 1.91 s. This demonstrates its ability to dynamically adjust to unexpected obstacles. These results validate the system’s ability to operate efficiently in real-world-like environments while highlighting areas for improvement, particularly in scalability and real-time adaptation.

**TABLE 2:** Quantitative evaluation of the proposed method.

Metric	2D Simulation	3D Gazebo
	(Test 1)	(Test 2)
Object Detection Accuracy	95%	92%
Collision Avoidance Success Rate	100%	90%
Path Following Error (m)	0.15 m	0.20 m
Path Efficiency	94%	90%
Scalability (Number of Robots)	8 Robots	6 Robots
Real-Time Adaptation (Path Re-routing Speed)	-	1.91 s per re-route

The evaluation format presented in Table 2 provides a structured comparison of key features across various multi-robot collaborative manipulation frameworks. While this format may not be universally standardized in the literature, it captures critical aspects such as control architecture, obstacle handling, object detection methods, robotic manipulator capabilities, and path planning algorithms. These attributes were selected based on their relevance to the system’s performance and adaptability in dynamic environments. Our choice of evaluation metrics—Collision Avoidance Success Rate, Path Following Error, and Path Efficiency—was guided by the need to assess the system’s ability to operate effectively in cluttered and unpredictable scenarios. For instance, the Path Following Error metric reflects the precision of the Pure Pursuit controller in maintaining trajectories, while Path Efficiency evaluates the system’s ability to navigate optimally despite environmental challenges. The Collision Avoidance Success Rate highlights the robustness of our real-time object avoidance algorithm.

Direct comparisons with results from similar studies are challenging due to differences in the experimental environments. Our framework operates within the Gazebo environment, which offers unique advantages for prototyping but may not fully replicate the conditions of physical experiments. Second, the robotic platforms used in this study (HUSKY mobile robots and KINOVA Gen3 manipulators) differ from those employed in other research, making apples-to-apples comparisons impractical. Additionally, many of the methods lack publicly available implementations or detailed descriptions of their testing protocols, precluding attempts to reproduce their results under identical conditions.

In addition to the discussed metrics, another important consideration is the system’s responsiveness to dynamic obstacles with varying motion profiles. In this work, the speed of all mobile robots and dynamic obstacles, including simulated humans, was set to 1.5 m/s (5 km/h). At this relatively low speed, the system has sufficient time to detect, process, and respond to dynamic changes in the environment. This ensures that the robots can effectively execute re-routing actions while maintaining stability and coordination during collaborative tasks. The choice of this speed aligns with typical operational velocities in warehouse and industrial settings, where safety and precision are prioritized over high-speed navigation. However, the system was not explicitly evaluated under high-speed conditions in this study. High-speed scenarios introduce additional challenges, such as reduced reaction times and increased computational demands for real-time decision-making. Addressing these challenges is critical for deploying the system in more demanding environments, such as urban or fast-paced logistics operations. Therefore, future work will focus on evaluating system performance under high-speed conditions, both in simulation and in real-world deployments. This will involve fine-tuning parameters such as detection thresholds, re-planning frequencies, and control gains to ensure robustness and stability.

To substantiate the findings of our simulation study, the next phase of our work involves deploying the proposed multi-robot collaborative manipulation framework in a real-world environment using physical robotic hardware. The experimental setup will replicate the sensor configurations used in the Gazebo simulation and integrate mobile robots with robotic manipulators. The primary objective is to assess the system’s robustness under varying operational conditions, including environments with both static and dynamic obstacles. The experimental validation will be structured as follows:

• Real-Time Performance Assessment: The system’s real-time capabilities will be evaluated by comparing key performance metrics between the simulation and physical implementation. This analysis will include:
  • Trajectory tracking accuracy: Measuring deviations between planned and executed paths.
  • Object detection performance: Evaluating detection rates and classification accuracy in dynamic conditions.
• Evaluation Scenarios: The framework will be tested under multiple operational scenarios to assess its adaptability and effectiveness:
  1. Static Obstacle Avoidance: Robots will navigate predefined paths in an environment with fixed obstacles, ensuring efficient path planning and collision-free operation.
  2. Dynamic Obstacle Avoidance: The system will be challenged with moving obstacles, such as human operators or autonomous agents, to evaluate its ability to respond to unpredictable changes in the environment.
  3. Collaborative Manipulation Tasks: Multi-robot teams will perform complex object transportation tasks (e.g., cooperative lifting and moving of large objects) under varying coordination constraints. These experiments will test synchronization capabilities and system adaptability to unexpected perturbations.
• Performance Metrics: The evaluation criteria will include:
  • Path-following accuracy and deviation analysis.
  • Task execution time and efficiency.
  • Object detection accuracy in real-time environments.
  • Collision rates and obstacle avoidance success rate.
  • Scalability assessment by varying the number of collaborating robots.

The results from the physical deployment will be compared with simulation outcomes to identify discrepancies and refine the system’s parameters, accounting for real-world uncertainties such as sensor noise and communication delays. This real-time validation will provide critical insights into the practical deployment feasibility of the proposed framework, ensuring its resilience and adaptability in dynamic, unstructured environments.

## 6 Conclusion

This work successfully demonstrates a robust approach to multi-robot collaboration and autonomous navigation in dynamic, obstacle-rich environments, achieved through the integration of Gazebo simulation, MATLAB, and Simulink. Using a combination of ROS, Simulink’s Subscribe and Publish blocks, and advanced control techniques such as Pure Pursuit and RRT-based path planning, the system facilitated flexible and adaptive real-time navigation, object detection, and obstacle avoidance. Experimental results in a collaborative transport task, where a team of Husky-Kinova pairs of robots transported a large object while avoiding both static and dynamic obstacles, confirmed the system’s ability to perform complex tasks in coordination, without collisions, even with humans and warehouse robots moving unpredictably in the environment.

The main contributions of this work include dynamic obstacle handling, multi-robot coordination, and scalability and flexibility. The object detection and avoidance algorithm efficiently processed sensor inputs, enabling seamless navigation among dynamic obstacles such as humans and automated warehouse vehicles, while each robot synchronized its actions through continuous feedback from Simulink’s Subscribe block and command updates via the Publish block to maintain alignment and stability during collaborative operations. Additionally, the approach demonstrated scalability across various multi-robot configurations, with parameter adjustments in Simulink and ROS allowing for dynamic and adaptable team setups.

These findings have significant implications for real-world applications, such as automated warehousing, industrial material handling, and logistics, where collaborative multi-robot teams can improve efficiency and safety. Additionally, the capability for real-time path adaptation and synchronized control holds promise for deploying MRS in unpredictable environments.

Future work will focus on deploying the developed logic and algorithms onto physical hardware in real-world scenarios. Implementing these strategies on actual robots as a stand-alone ROS node will allow for validation of the system’s robustness outside the simulation environment, offering insights into potential challenges and adjustments required for real-time performance. This deployment will also provide opportunities to enhance the algorithms to handle real-world factors, such as sensor noise, complex terrain, and communication delays, further advancing the system’s utility in practical multi-robot applications.

## Data Availability

The original contributions presented in the study are included in the article/supplementary material, further inquiries can be directed to the corresponding author.
